# Lower ghrelin levels does not impact the metabolic benefit induced by Roux-en-Y gastric bypass

**DOI:** 10.3389/fendo.2022.891379

**Published:** 2022-08-23

**Authors:** Yuan Liang, Ruili Yu, Rui He, Lijun Sun, Chao Luo, Lu Feng, Hong Chen, Yue Yin, Weizhen Zhang

**Affiliations:** ^1^ Department of Physiology and Pathophysiology, School of Basic Medical Sciences, and Key Laboratory of Molecular Cardiovascular Science, Ministry of Education, Peking University, Beijing, China; ^2^ Department of Pathology, Henan Provincial People’s Hospital; People’s Hospital of Zhengzhou University, Zhengzhou, China; ^3^ Key Laboratory of Fertility Preservation and Maintenance of Ministry of Education, School of Basic Medical Sciences, Ningxia Medical University, Yinchuan, China; ^4^ Department of Surgery, University of Michigan Medical Center, Ann Arbor, MI, United States

**Keywords:** Roux-en-Y gastric bypass, ghrelin, body weight, glucose metabolism, lipid metabolism

## Abstract

**Objective:**

Roux-en-Y gastric bypass is an effective intervention for metabolic disorder. We aim to elucidate whether ghrelin contributes to weight reduction, and glycemic and lipid control after Roux-en-Y gastric bypass (RYGB).

**Design:**

Four-week-old WT and Ghrl-TSC1^-/-^ mice were fed high fat diet for 12 weeks before surgery, and continued to be on the same diet for 3 weeks after surgery. Body weight, food intake, glycemic and lipid metabolism were analyzed before and after surgery.

**Results:**

Gastric and circulating ghrelin was significantly increased in mice with RYGB surgery. Hypoghrelinemia elicited by deletion of TSC1 to activate mTOR signaling in gastric X/A like cells demonstrated no effect on weight reduction, glycemic and lipid control induced by Roux-en-Y gastric bypass surgery.

**Conclusion:**

Lower ghrelin levels does not impact the metabolic benefit induced by Roux-en-Y gastric bypass.

## Introduction

Metabolic syndrome is characterized by obesity, hypertension, insulin resistance, and hyperlipidemia. According to CDC data published in 2017, about one third of US adults have metabolic disorders (1).

Bariatric surgery, in particular, the Roux-en-Y gastric bypass (RYGB), is an effective intervention to weight reduction for metabolic disorders (2). The metabolic benefits of RYGB include substantial weight reduction and a significant improvement in glycemic control. This surgery results in alteration in structure and physiological function of the gastrointestinal tract, including changes in the production and secretion of gastrointestinal hormones (3). Transgenic mice have been used to investigate the role of gut hormones in the metabolic phenotypes of bariatric surgery. For example, the effects of bariatric surgery on improvement of glucose tolerance and lipid metabolism also were maintained in ghrelin KO mice (4). GLP-1R was found not necessary for the enhanced glucose control induced by Vertical Sleeve Gastrectomy (VSG) (5). In a global PYY receptor (Y2R) knockout mouse model, PYY signaling was found not necessary for the normal appetite suppression and weight loss effects of RYGB (6). However, whether weight reduction and the improvement of glycemic and lipid control after bariatric surgery depends on gastrointestinal hormones is still under debate.

Ghrelin, a unique 28 amino acid peptide, is well known for its appetite-stimulating action *via* activation of its receptor, the growth hormone secretagogue receptor 1a (GHSR1a) (7). This hormone is mainly produced by X/A like cells, the second most abundant gastroendocrine cells located in fundus of stomach (8). The main physiological functions of ghrelin include regulation of weight gain, appetite, lipid metabolism and glucose metabolism (9–12). Levels of circulating ghrelin are negatively correlated with body mass index and regulated by alterations of body weight (13–16). Chronic ghrelin administration induces weight gain by stimulating food consumption and suppressing fat catabolism (17–19). In previous study, we have demonstrated that X/A like cells sense organism energy levels through the mTOR signaling to regulate the production and secretion of ghrelin (11), which in turn contributes to the control of glucose and lipid homeostasis (20, 21). Further, circulating levels of ghrelin have been demonstrated to be either decreased or increased after bariatric surgery (22–25). This discrepancy has raised the debate on whether (a) ghrelin may contribute to metabolic benefit induced by bariatric surgery; and (b) change in ghrelin is a consequence or cause for weight control induced by bariatric surgery.

Here, we reported that RYGB increased ghrelin translation and secretion. Lower ghrelin levels induced by deficiency of tuberous sclerosis complex 1 (TSC1) in X/A like cells of Ghrl-TSC1^-/-^(TG) transgenic mice, demonstrated no effect on body weight reduction, as well as glycemic and lipid control after RYGB surgery.

## Materials and methods

### Animals

Animal experiments were performed in strict accordance with the Guide for the Care and Use of Laboratory Animals (NIH publication 86-23, revised 1985). All experimental protocols were approved by the Animal Care and Use Committee of Peking University. Four-week-old C57BL/6J male mice were purchased from the Department of Experimental Animal Science, Peking University Health Science Center. *Ghrl-TSC1* ^-/-^ (TG) mice, X/A like cells-specific *TSC1* knockout mice, with the C57BL/6J genetic background were generated by crossing *Ghrl-Cre* mice with *TSC1^loxp/loxp^* mice, which were purchased from the Jackson Laboratory. The genotype identification and use of TG mice has been mentioned in our previous studies (20, 21). Mice were housed in standard plastic rodent cages and maintained in SPF environment. Normal chow diet or high fat diet and water were available ad libitum unless specified fasting experiment.

Four weeks-old WT and TG mice were fed a high-fat diet (HFD) (60% fat, D12492; Research Diets) or a normal chow diet (NCD) (control diet, D12450H; Research Diets) for12 weeks. At the time of surgery, WT or TG mice were divided into Sham and RYGB groups randomly. Mice were fed with HFD or NCD for 3 weeks after surgery until the end of experiments. At the end of the experiment, following tissue samples were harvested from mice: plasma, stomach, liver, epididymal white adipose tissue (eWAT), subcutaneous white adipose tissue (sWAT) and brown adipose tissue (BAT).

### Surgical procedures

RYGB Surgery and the sham operation were performed as described previously (26). Standard aseptic procedures were applied throughout.

### Measurement of plasma ghrelin and triglyceride

After anesthesia, blood samples were transcardially collected between 8-10am from mice fed ad libitum. Plasma was separated by centrifugation at 1600g for 15 min at 4°C and stored at − 80°C. According to the manufacturer’s instructions, total ghrelin levels in blood were measured by enzyme linked immunosorbent assay (ELISA). Acylated ghrelin was measured using radio immunoassay kit (Linco Bioscience Institute, St. Charles, MO).

Liver was homogenized with 1 ml of a 2:1 chloroform and methanol mixture at 4°C and stored at 4°C overnight. The homogenate was mixed with 200ul double-distilled water, and centrifuged at 3000 rpm for 10 min at 4°C. The lower phase was collected, vacuum dried and dissolved in 5% Triton X-100. Levels of liver and plasma triglyceride was measured according to the kit’s instructions (Nanjing Jiancheng Bioengineering Institute, Nanjing, China).

### Glucose metabolism

#### Oral glucose tolerance tests

Mice fasted for 16 hours were orally gavaged with glucose at a dose of 3 g/kg body weight. Blood was collected at the tip of the tail at 0, 15, 30, 60, 90, and 120 minutes after glucose administration, and the glucose concentration measured.

#### Insulin tolerance test

Mice were fasted for 6 hours before intraperitoneal insulin administration at a dose of 1U/kg body weight. Blood was collected at the tip of the tail at 0, 15, 30, 60, 90, and 120 minutes after insulin administration, and the glucose concentration measured immediately.

### Tissue sample preparation and histological examination

Mice were anesthetized using pentobarbital (0.07 g/kg). Liver and adipose tissues were quickly removed and then fixed in fixative, embedded, sectioned and adhered to charged slides. The sections were used for H&E and Oil Red O staining following general protocols.

### Analysis of gene expression

Total RNA was isolated from stomach, liver and adipose tissues using Trizol, followed by reverse transcription. PCR amplification was conducted on an AriaMx Real-Time PCR system (Agilent Technologies, CA, USA) by a mixture containing upstream and downstream primers, Hieff qPCR SYBR Green Mater Mix, and cDNA template. *β-actin* was used as internal control. Primer sequences were provided in **Table S1**.

### Statistical analysis

All data were shown as mean ± SEM. Statistical significance of differences between the groups was analyzed with a t-test or one/two-way ANOVA, followed by Tukey multiple *post hoc* analysis. P<0.05 denotes statistical significance.

## Results

### Increment of ghrelin after RYGB

**Figure 1:**
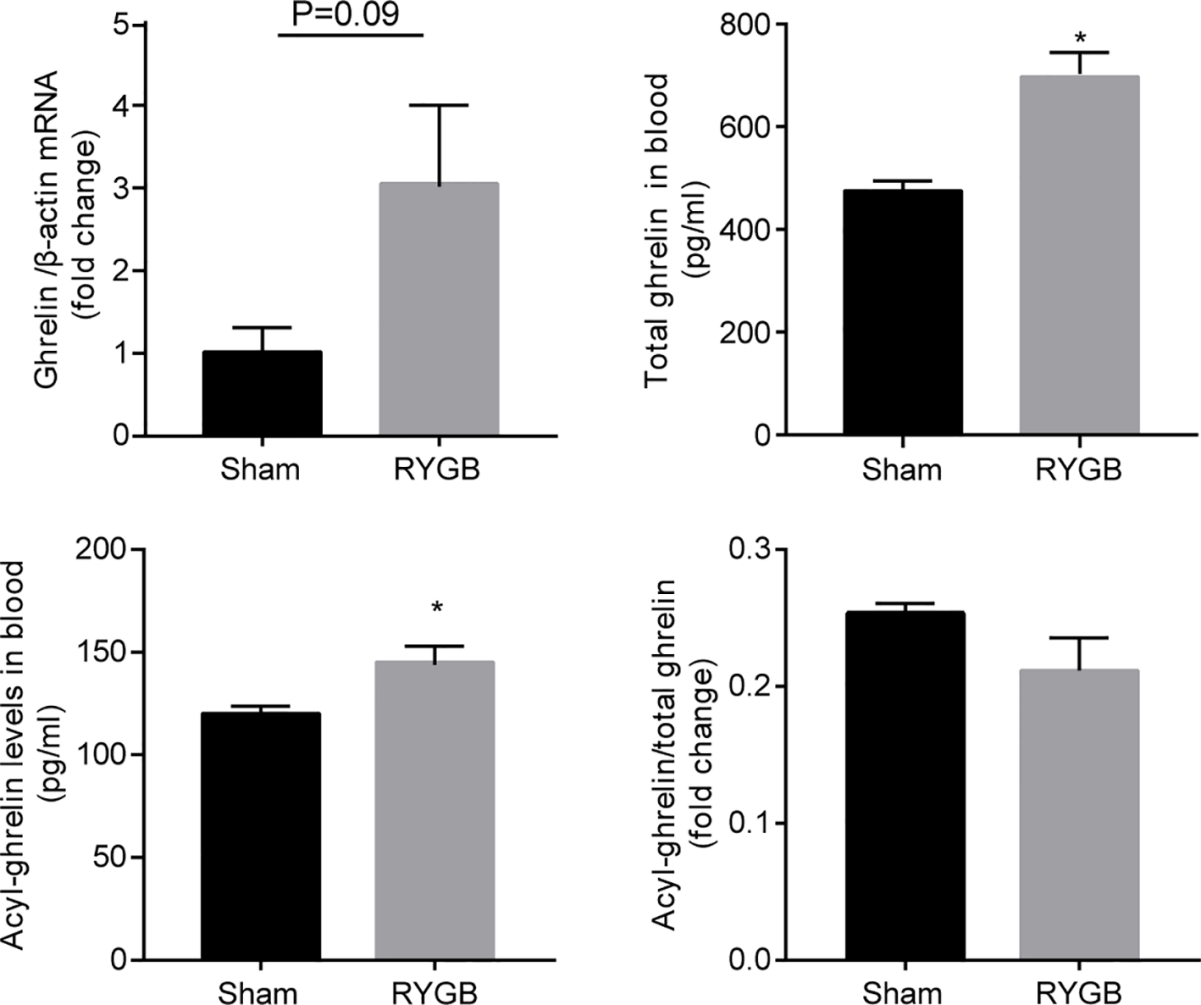
Increment of ghrelin after RYGB C57BL/6J mice were fed with high fat diet (HFD) for 12 weeks prior to and 3 weeks after Roux-en-Y gastric bypass surgery (RYGB). mRNA levels of gastric *Ghrelin*, and plasma total ghrelin and acyl-ghrelin were measured and the ratio of acyl-ghrelin/total ghrelin calculated. *β-actin* was used as internal control. Data was presented as mean ± SEM. P values were determined by unpaired t test. ^*^P<0.05 vs. sham. n=4 mice per group.

To determine whether ghrelin contributes to weight control by RYGB, we first examined the alteration in levels of ghrelin after RYGB surgery. As shown in **Figure 1**, RYGB significantly increased levels of gastric *ghrelin* mRNA as well as plasma acyl-ghrelin and total ghrelin relative to sham-operated mice. Interestingly, the ratio of acyl ghrelin/total ghrelin remained largely unchanged.

### Lower ghrelin levels in ghrl-TSC1^-/-^ mice has no effect on food intake and weight reduction after RYGB

**Figure 2:**
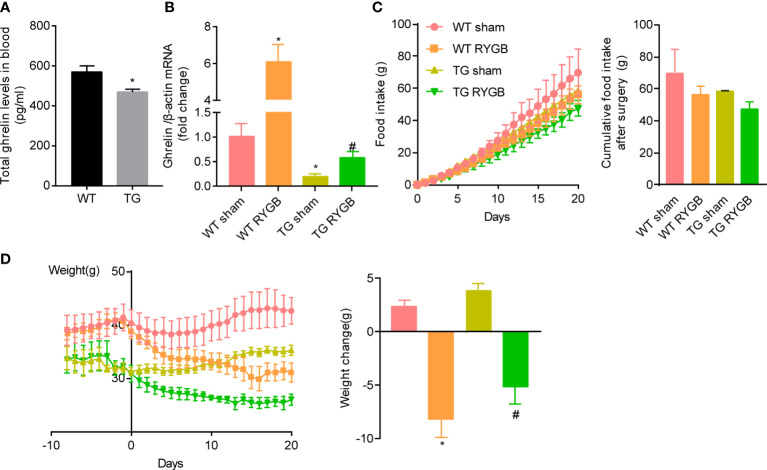
Effect of RYGB on food intake and body weight in TG mice with lower ghrelin levels *Ghrl-TSC1* ^-/-^ (TG) transgenic mice were generated by cross-breeding *Ghrl-cre* transgenes with TSC1 ^flox/flox^ mice. Validated TG mice and wild-type littermates (WT) were fed with high fat diet (HFD) for 12 weeks before surgery and 3 weeks after surgery. Results were expressed as mean ± SEM. P values were determined by unpaired t test or one-way ANOVA, followed by Tukey multiple *post hoc* analysis. ^*^P<0.05 vs. WT Sham. ^#^P < 0.05 vs. TG Sham. **(A)** Plasma total ghrelin (n=6 and 7 for WT and TG respectively). **(B)** mRNA levels of gastric *Ghrelin* in WT or TG mice with sham or RYGB surgery. *β-actin* was used as internal control (n=4 mice per group). **(C)** Cumulative food intake. **(D)** Body weight and weight change after RYGB surgery (n=6 for WT sham, 4 for WT RYGB, 4 for TG sham, and 4 for TG RYGB).

The observation that ghrelin production and secretion are increased after RYGB surgery promoted us to determine whether ghrelin contributes to the metabolic benefit induced by this surgery using the hypoghrelinemic ghlr-TSC1^-/-^ (TG) mice. Deletion of TSC1 in X/A like cells and activation of gastric mTOR signaling by deletion of TSC1was confirmed by genotyping and immunofluorescence (20, 21). Activation of gastric mTOR signaling induced lower ghrelin levels in TG mice (**Figure 2A**). This reduction in plasma ghrelin level in TG mice was consistent with our previous reports.

To functionally assess the role of ghrelin on food intake and body weight control induced by RYGB, TG and wild-type littermates (WT) mice were fed 60% high fat diet for 12 weeks to induce obesity. At the time of surgery, obese mice were randomly divided into sham and RYGB groups. As shown in **Figure 2B**, mRNA levels of gastric *ghrelin* were significantly reduced in TG mice with either sham or RYGB surgery. Increment in gastric *ghrelin* mRNA induced by RYGB surgery was significantly attenuated in TG mice. Despite of the reduction in ghrelin production, TG mice with RYGB surgery demonstrated no significant difference in cumulative food intake (**Figure 2C**) relative to WT mice with RYGB surgery. These results indicate that lower ghrelin levels in TG mice is not sufficient to attenuates the effect of RYGB on food intake. Further, RYGB was able to reduce body weight in TG mice with deficiency of ghrelin to an extent comparable to that in wild type mice (**Figure 2D**). Interestingly, both food intake and body weight were significantly reduced in ghrelin-deficient TG mice with either sham or RYGB surgery. All these results indicate that ghrelin is not the only factor for reduction in food intake and body weight induced by RYGB surgery.

### High levels of ghrelin is dispensable in glycemic control after RYGB

**Figure 3:**
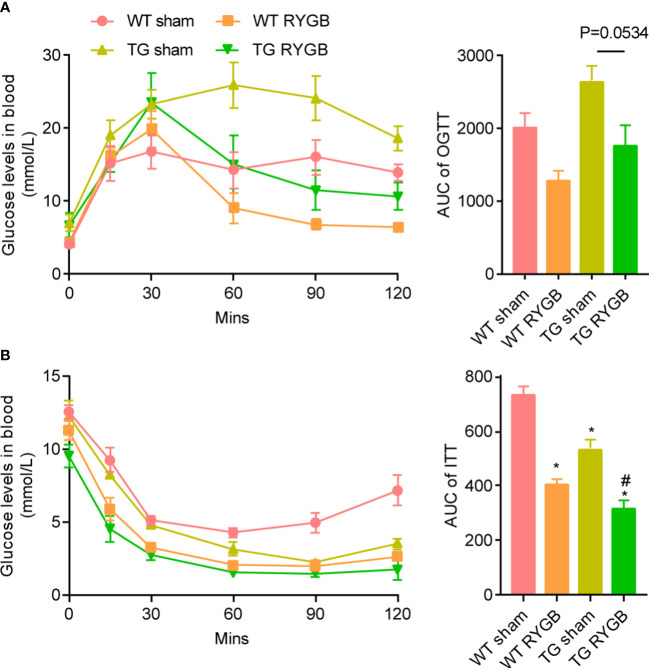
Effect of RYGB on glycemic control in TG mice with lower ghrelin levelsValidated TG mice and wild-type littermates were fed with high fat diet (HFD) for 12 weeks before surgery and 3 weeks after surgery. Results were expressed as mean ± SEM. P values were determined by one-way ANOVA, followed by Tukey multiple *post hoc* analysis. ^*^P<0.05 vs. WT Sham. ^#^P<0.05 vs. TG Sham. n=4 mice per group. **(A)** Oral glucose tolerance test and the area under curve. **(B)** Insulin tolerance test and the area under curve.

RYGB induced similar improvement in glucose metabolism measured by oral glucose tolerance (**Figure 3A**) and insulin sensitivity test (**Figure 3B**) in TG mice relative to WT mice.

### Ghrelin is not the sole player in the improvement of adipocyte browning after RYGB

**Figure 4:**
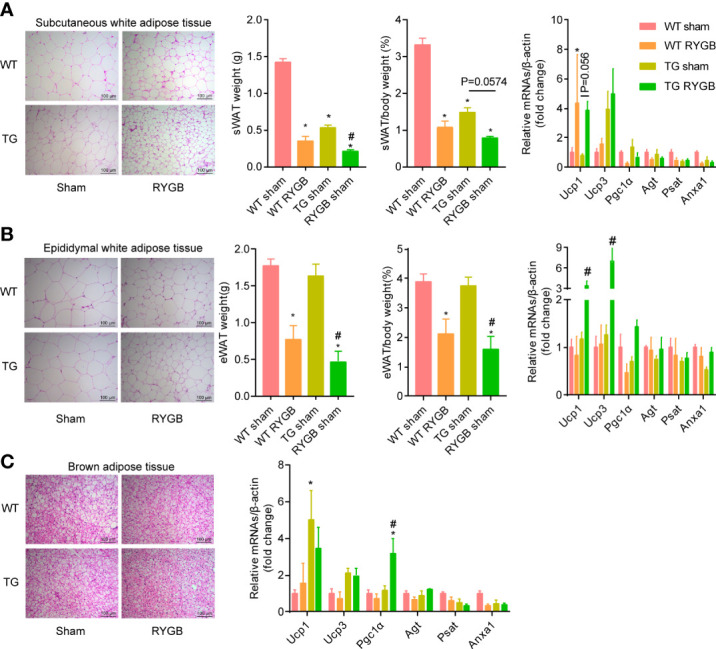
Effect of RYGB on white adipose tissue beigeing in TG mice with lower ghrelin levels Validated TG mice and wild-type littermates (WT) were fed with high fat diet (HFD) for 12 weeks before surgery and 3 weeks after surgery. Shown were histological (H&E) morphology, tissue weight, mRNA levels of brown-adipocyte marker genes (*Ucp1, Ucp3, Pgc1α*) and white-adipocyte marker genes (*Agt, Psat, Anxa1*) of sWAT **(A)**, eWAT **(B)** and BAT **(C)** of WT or TG mice. Results were expressed as mean ± SEM. P values were determined by one/two-way ANOVA, followed by Tukey multiple *post hoc* analysis.^*^P<0.05 vs. WT Sham. ^#^P<0.05 vs. TG Sham. n=4-6 mice per group.

To determine whether ghrelin contributes to the increment in adipocyte browning after RYGB, we examined the change in subcutaneous and visceral white adipose tissues in TG mice. Adipocyte size and adipose tissue weight in subcutaneous white adipose tissue (**Figure 4A**) and epididymal white adipose tissue (**Figure 4B**) were reduced to a similar extent in WT and TG mice with RYGB. Further, mRNA levels of brown-adipocyte marker gene *Ucp1* in subcutaneous white adipose tissues were significantly increased in TG mice with RYGB surgery. In brown adipose tissue (BAT), RYGB induced identical morphological change in TG mice relative to wild type mice (**Figure 4C**). Lipid droplet vacuolization was significantly decreased, whereas oxyphilic structure increased. Further, RYGB surgery elicited a significant increase of *Pgc1α* mRNA in TG mice relative to sham group. Interestingly, TG mice demonstrated a marked reduction in subcutaneous white adipose tissues.

### High levels of ghrelin is dispensable in amelioration of liver steatosis after RYGB

**Figure 5:**
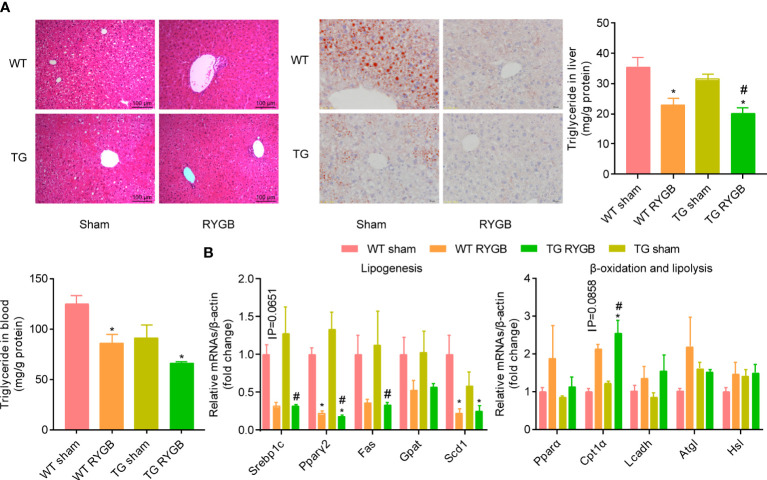
Effect of RYGB on liver steatosis in TG mice with lower ghrelin levels Validated TG mice and wild-type littermates (WT) were fed with high fat diet (HFD) for 12 weeks before surgery and 3 weeks after surgery. Results were expressed as mean ± SEM. P values were determined by one/two-way ANOVA, followed by Tukey multiple *post hoc* analysis. ^*^P<0.05 vs. WT Sham. ^#^P<0.05 vs. TG Sham. n=4-6 mice per group. **(A)** Liver histology (H&E), Oil red staining, hepatic and plasmic contents of triglyceride. **(B)** mRNA levels of hepatic lipogenesis, β-oxidation and lipolysis marker genes.

To evaluate whether ghrelin contributes to improvement in liver steatosis after RYGB, H&E and Oil Red O staining, triglyceride in liver and blood (**Figure 5A**), mRNA levels of lipogenesis, β-oxidation and lipolysis marker genes (**Figure 5B**) were assayed. As showed in **Figure 5A**, RYGB decreased hepatic lipid drops, levels of triglyceride in liver in WT mice. Similar changes were observed in TG mice. Levels of lipogenesis-related transcriptional factors (*Srebp1c* and *Pparγ2*) were decreased after RYGB, regardless of TG or wild type mice. Meanwhile, hepatic β-oxidation related marker gene *Cpt1α* was increased after RYGB in either wild type or TG mice (**Figure 5B**).

## Discussion

A series of physiological mechanisms have been proposed to contribute to the metabolic benefit of bariatric surgery (27). Among these mechanisms are enteroplasticity, neurophysiological adaptation, changes in gut hormones, signaling in bile acids and relevant receptors such as Farnesoid X receptor (FXR) and Takeda G protein-coupled receptor 5 (TGR5), and gut microbiota and metabolites, as well as many other yet-to-be identified mechanisms (28, 29). Evidence has been emerging that the overall metabolic benefit following bariatric surgery is attributed to the inter-dependent connection of these mechanisms, rather than a single player (30–32). For example, several gut hormones such as ghrelin (4) and glucagon like peptide 1 (GLP-1) (33)have been reported to be dispensable for the long-lasting decrement of appetite and sustainable body weight control after bariatric surgery. Consistently, our studies provide evidence that ghrelin is not the sole mechanism contributing to food intake and body weight reduction, as well as glycemic and lipid control after RYGB.

Firstly, RYGB surgery leads to the higher ghrelin levels in both lean and diet-induced-obese mice. Both the translation and secretion of ghrelin increase after RYGB. This alteration is contradicting the reported function for ghrelin to increase the energy conservation. Thus, the increment in circulating ghrelin occurs likely as a consequence of adaptive response to the RYGB surgery. Change in circulating ghrelin levels has been conflictingly reported to be either increased, unaltered or decreased. Reason underlying this controversy is unclear but may be related to type of surgery, sample processing and postoperative period. Postoperative ghrelin levels decrease significantly after sleeve gastrectomy (34). Since majority of circulating ghrelin is derived from the X/A like cells located in the gastric fundus, removal of large portion of stomach in sleeve gastrectomy thus results in prolong reduction in both fasting and postprandial ghrelin levels. On the other hand, RYGB may relieve the inhibition of ghrelin secretion induced by food intake, leading to subsequent increase in ghrelin production.

Secondly, lower ghrelin levels induced by knocking down TSC1 to activate gastric mTOR signaling results in reduction of food intake and body weight, and improvement in lipid metabolism. However, TG mice show an impaired glucose metabolism which is attributed to the pancreatic fibrosis as reported in our previous study (21). Ghrelin has been consistently demonstrated to stimulate food intake, but its effects on body weight, glucose and lipid metabolism have been controversial. The controversy may be attributed to the interconnection of various physiological functions of ghrelin. For example, ghrelin may induce weight gain and subsequent metabolic dysfunction. However, ghrelin has been shown to potently suppress inflammation. Thus, this hormone may reduce the chronic metabolic inflammation relevant to obesity, leading to subsequent improvement in metabolic dysfunction. Indeed, our previous studies have shown that ghrelin may directly act on hepatocytes to increase lipogenesis and lipid deposit in liver under physiological condition (11). In contrast, ghrelin potently inhibits M1 polarization of macrophages, leading to subsequent amelioration of steatohepatitis induced by low dose of lipopolysaccharides in diet-induced-obese mice (35). Spontaneous pancreatic fibrosis occurs in hypoghrelinemic TG mice, indicating an anti-fibrosis function of ghrelin. Severe pancreatic fibrosis reduces the secretion of insulin which may jeopardize the glucose metabolism. Further investigation should aim to reveal the cell specific effect of ghrelin by using the conditional genetic manipulation of ghrelin receptor. Further, previous studies found that RYGB promotes white adipose tissue browning (36) and reduction in adipose size, as well as up-regulated expression of brown adipose markers in adipose tissues was observed in TG mice (20). As a result, enhanced white fat browning was observed in TG RYGB mice.

Thirdly, the metabolic benefits induced by RYGB surgery such as reduction in food intake and body weight, as well as improvement in glycemic and lipid control, are persisted in TG mice with lower ghrelin levels. This observation again indicates the complex mechanisms by which bariatric surgery improves metabolic dysfunction. Understanding these inter-connected pathways is of great significance for dissecting the pathogenesis of metabolic diseases, as well as providing novel non-surgical intervention of obesity.

In conclusion, our studies indicate that ghrelin is not the sole mechanism contributing to the metabolic benefit of Roux-en-Y gastric bypass surgery. Our findings further highlight the strategy of targeting multiple pathways for efficient intervention of obesity and its relevant metabolic dysfunction.

## Data availability statement

The raw data supporting the conclusions of this article will be made available by the authors, without undue reservation.

## Ethics statement

The animal study was reviewed and approved by Animal Care and Use Committee of Peking University.

## Author contributions

YL: data interpretation and manuscript drafting; RY: data acquisition and analysis, funding support; RH: surgical operation; YY and WZ: experiment design, manuscript revision, funding support; LS, CL, LF and HC: technical and reagents support. All other authors edited and approved the final manuscript.

## Funding

This study was supported by National Natural Science Foundation of China (81730020, 81930015, 82070592, 82100923) and National Institutes of Health Grant R01DK112755, 1R01DK129360 and 1R01DK110273.

## Conflict of interest

The authors declare that the research was conducted in the absence of any commercial or financial relationships that could be construed as a potential conflict of interest.

## Publisher’s note

All claims expressed in this article are solely those of the authors and do not necessarily represent those of their affiliated organizations, or those of the publisher, the editors and the reviewers. Any product that may be evaluated in this article, or claim that may be made by its manufacturer, is not guaranteed or endorsed by the publisher.

## Abbreviations

RYGB, Roux-en-Y gastric bypass; GHSR, growth hormone secretagogue receptor; TG, ghrl-TSC1−/−; HFD, high-fat diet; eWAT, epididymal white adipose tissue; sWAT, subcutaneous white adipose tissue; BAT, brown adipose tissue; Elisa, enzyme linked immunosorbent assay; OGTT, oral glucose tolerance tests; ITT, insulin tolerance test.
