# Supplementary material for: Lower ghrelin levels does not impact the metabolic benefit induced by Roux-en-Y gastric bypass
Source: Front Endocrinol (Lausanne). 2022 Aug 23;13:891379. doi: 10.3389/fendo.2022.891379 (PMC9445200; doi:10.3389/fendo.2022.891379)
Supplement: Supplementary file 1 [file Table_1.docx]

Table S1

Primers for Quantitative RT-PCR

| **Genes** | **Upstream primer (5’-3’)** | **Downstream primer (5’-3’)** |
| --- | --- | --- |
| Ghrelin | CCATCTGCAGTTTGCTGCTA | GCAGTTTAGCTGGTGGCTTC |
| Ucp1 | GGACGACCCCTAATCTAATG | CATTAGATTAGGGGTCGTCC |
| Ucp3 | ATCAGGATTCTGGCAGGC | GCCTGCCAGAATCCTGAT |
| Pgc1a | GATTGAAGTGGTGTAGCGAC | GTCGCTACACCACTTCAATC |
| Agt | GGAGTGACACCCAGAACA | TAGATGGCGAACAGGAAG |
| Psat | CGGAGATCAACTTTCTGG | TGGAGACGATTTCAATGG |
| Anxa1 | CCCTTCCTTCAATGTATCC | GCATAGCCAAAACAACCTC |
| Srebp1c | GGAGCCATGGATTGCACATT | GGAAGTCACTGTCTTGGTTGTTGA |
| Pparγ2 | CTCTGGGAGATTCTCCTGTTGA | GGTGGGCCAGAATGGCATCT |
| Fas | TGGGTTCTAGCCAGCAGAGT | ACCACCAGAGACCGTTATGC |
| Gpat | CACACGAGCAGGAAAGATGA | GGACTGCATAGATGCTGCAA |
| Scd1 | GCGATACACTCTGGTGCTCA | CCCAGGGAAACCAGGATATT |
| Pparα | GAGAAGTTGCAGGAGGGGATTGTG | AAGACTACCTGCTACCGAAATGGG |
| Cpt1α | ATCGTGGTGGTGGGTGTGATAT | ACGCCACTCACGATGTTCTTC |
| Lcadh | CTCCCTGCGCGTCCTGAG | AAAATGTCATGCTCCGAGGAAAAG |
| Atgl | AACACCAGCATCCAGTTCAA | GGTTCAGTAGGCCATTCCTC |
| Hsl | GATTTACGCACGATGACACAGT | ACCTGCAAAGACATTAGACAGC |
| β-actin | ATCTGGCACCACACCTTC | AGCCAGGTCCAGACGCA |
